# Behavioral pathways and psychosocial determinants of adolescent mental health: positioning nutrition health literacy as a candidate upstream target

**DOI:** 10.3389/fnut.2026.1908234

**Published:** 2026-08-26

**Authors:** Wang Tuo, Wang Sicong, Lyu Wei

**Affiliations:** 1 School of General Education, Xihang University, Xi’an, China; 2 School of Teacher Education, Hebei Normal University, Shijiazhuang, China; 3 Faculty of Medicine and Health, Al-Farabi Kazakh National University, Almaty, Kazakhstan

**Keywords:** adolescents, dietary behavior, eating disorders, health promotion, mental health, nutrition health literacy, school-based intervention

## Abstract

Adolescence is a sensitive window for both nutritional and psychological development, and mental health problems in this group are rising worldwide. Nutrition health literacy, the capacity to obtain, understand, appraise, and apply nutrition information, has been proposed as a modifiable upstream factor that may connect dietary behavior with mental health, although evidence directly linking nutrition health literacy to mental health outcomes remains scarce. This mini review therefore synthesizes recent evidence on the behavioral and psychosocial routes to adolescent mental health, and asks how far nutrition health literacy can currently be supported as an upstream target. We first examine the multidimensional nature of nutrition health literacy, its boundaries with food literacy and mental health literacy, and the difficulty of measuring it consistently in young people. We then describe three behavioral pathways: diet, physical activity, and sleep, through which nutrition health literacy may shape emotional outcomes. Unhealthy eating patterns, such as skipping breakfast and high intake of sugar-sweetened beverages, are consistently associated with greater risks of anxiety and depression in cross-sectional work, whereas prospective cohort evidence is smaller and less consistent. We next consider psychosocial determinants operating at the family, peer, school, and sociocultural levels, which together form the ecological context of adolescent behavior. We review school- and community-based prevention programs, which show small but meaningful effects that vary across subgroups and settings. Finally, we address current controversies, including debate over universal interventions, together with major research gaps in assessment standardization, longitudinal evidence, and representation of low- and middle-income countries. We argue for culturally sensitive, equity-oriented, and developmentally appropriate approaches. Strengthening nutrition health literacy is a plausible but as yet untested target for promoting adolescent mental health, and we set out the study designs needed to test it.

## 1 Introduction

Adolescence is a critical window for physical and psychological development. Nutritional status, dietary behavior, and mental health during this period shape not only current growth but also long-term health in adulthood. The prevalence of anxiety, depression, and eating disorders among adolescents continues to rise worldwide, posing a major public health challenge (1, 2). At the same time, unhealthy dietary patterns are common in this age group. These include skipping breakfast, excessive consumption of sugar-sweetened beverages, and inadequate intake of fruit and vegetables. Such patterns show complex associations with psychological distress and mood disorders (3, 4).

Against this background, nutrition health literacy, the capacity to obtain, understand, appraise, and apply nutrition information to make health-promoting decisions (5), has been proposed as an upstream factor connecting dietary behavior with mental health, and has been associated with dietary choices, physical activity, and health management in adolescents (6, 7). We stress at the outset, however, that the evidence below concerns diet and mental health far more than literacy itself; literacy as an intervening factor is plausible but rarely tested directly, and we treat it throughout as hypothesized rather than established (8). The title and structure of this review are framed accordingly: the behavioral and psychosocial evidence forms the substantive core, and nutrition health literacy is presented as a candidate upstream target that this evidence motivates but does not confirm.

Adolescents occupy a transitional stage, moving from dependence on the family toward autonomous decision-making. Parenting style, peer dietary norms, school health education, and media influence on body image together form a complex ecological system around their dietary behavior and psychological state (9, 10). School-based prevention programs, such as healthy lifestyle education, social and emotional learning, and peer education, show some promise. However, their effects differ markedly across studies and cultural settings, which has prompted debate about the generalizability of such strategies and their possible adverse effects (11–13).

**Figure 1:**
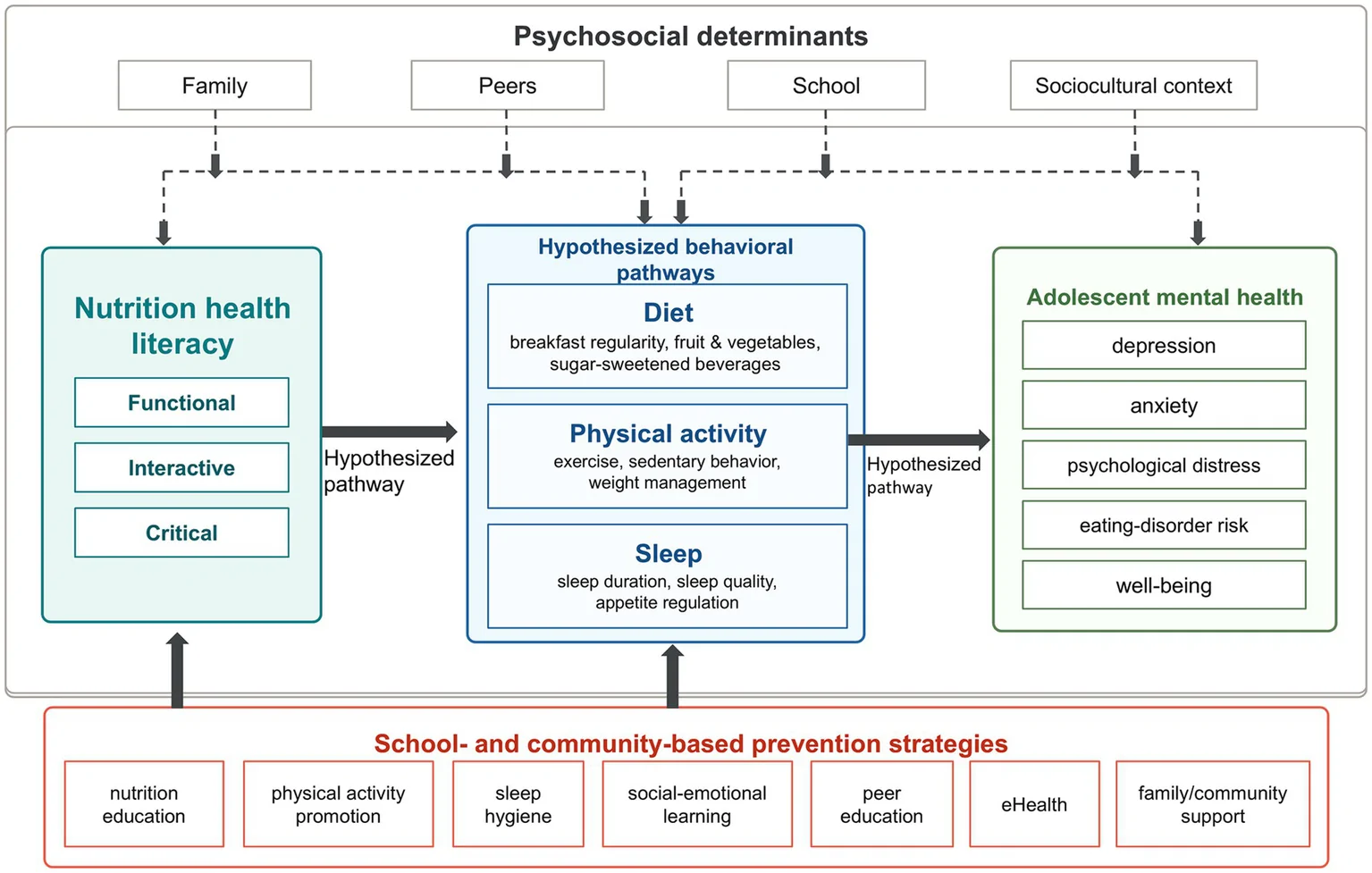
Conceptual framework linking nutrition health literacy to adolescent mental health. Nutrition health literacy is hypothesized to act through three interacting behavioral pathways—diet, physical activity, and sleep—to shape mental health outcomes. These relationships operate within an ecological context of psychosocial determinants and can be targeted by school- and community-based prevention. The model is a conceptual proposal that organizes the reviewed literature; the mediating role of nutrition health literacy shown here has not, to our knowledge, been directly tested, and the arrows denote hypothesized rather than empirically established effects.

This mini review therefore synthesizes evidence on the behavioral pathways, psychosocial determinants, and prevention strategies relevant to adolescent mental health, and evaluates how far nutrition health literacy can be positioned as an upstream target within them (Figure 1). That framework is an organizing heuristic rather than a validated causal model; to our knowledge, none of the studies reviewed formally tests nutrition health literacy as a mediator between diet and mental health.

### 1.1 Search strategy and scope

This is a narrative mini review, not a systematic review. We searched PubMed, Web of Science, and Scopus for English-language articles combining terms for adolescents with terms for nutrition or health literacy, dietary behavior, physical activity, sleep, and mental-health outcomes (depression, anxiety, eating disorders, psychological distress).

Recency was weighted for a specific reason rather than applied as a blanket rule. The literature on adolescent diet and mental health has expanded rapidly since 2023, and the purpose of a mini review is to map that dispersed new evidence rather than repeat existing syntheses; the emphasis on 2024–2026 therefore applied only to primary studies illustrating behavioral pathways, psychosocial determinants, and interventions. Two categories were exempt from any date restriction. Foundational conceptual and measurement work on health, nutrition, and food literacy was included irrespective of year (5, 14–19), precisely because studies of nutrition health literacy are scarce and most instrument development predates 2024, so a recency filter would have removed almost all of it. Longitudinal cohorts and earlier systematic reviews of diet and mental health were likewise retained regardless of year (8, 20–24), since prospective evidence is scarcer, more informative, and concentrated in earlier work.

Consistent with the format, we did not perform a formal quality appraisal, and studies are cited as illustrative rather than definitive. Table 1 summarizes the principal studies discussed, country, design, sample, constructs and instruments, and main findings, and records for each whether nutrition health literacy was itself measured; that last column is the most informative for present purposes. We return to these limitations in the Discussion.

**Table 1:** Characteristics of the principal studies discussed in this review.

Study	Country; design and sample	Constructs and instruments	Main findings relevant to this review	Nutrition health literacy measured?
Jacka et al. (21)	Australia; prospective cohort, 3,040 adolescents, 2-year follow-up	Diet quality (ARFS-derived index); psychological functioning (PedsQL)	Improved diet quality predicted improved psychological functioning; deterioration predicted the reverse, independent of baseline symptoms	No
Winpenny et al. (22)	UK; ROOTS prospective cohort, adolescents followed into late adolescence	Diet quality (food-frequency questionnaire); depressive symptoms (MFQ)	No cross-sectional or prospective association with depressive symptoms after adjustment for socioeconomic position, BMI, and health behaviors	No
Kandola et al. (23)	UK; ALSPAC prospective cohort, accelerometer-assessed activity, ages 12–16 to 18	Objectively measured physical activity and sedentary behavior; depressive symptoms (CIS-R)	Sedentary behavior predicted higher depressive symptoms at 18; light activity predicted lower symptoms	No
Orchard et al. (24)	UK; community adolescent sample, cross-sectional and prospective	Self-reported sleep patterns and quality; anxiety and depression	Reciprocal associations: poor sleep predicted later anxiety and depression, and baseline symptoms predicted later sleep problems	No
O’Neil et al. (8); Khalid et al. (20)	International; systematic reviews of children and adolescents	Dietary patterns and quality; depression and internalizing symptoms	Consistent direction of association, but evidence overwhelmingly cross-sectional; temporal ordering rarely established	No
Tucker et al. (1)	International; systematic review of adolescents	Dietary patterns; mental health outcomes	Unhealthy patterns associated with depressive and anxiety symptoms; healthy patterns with better outcomes	No
Pengpid et al. (3)	Philippines; national school-based survey (GSHS)	Meal skipping; dietary intake; psychological distress, suicidal ideation	68.1% skipped ≥1 meal in the past month; meal skipping associated with low fruit and vegetable intake, distress, and suicidal ideation despite 89.1% reporting healthy-eating education	No
Naumoska et al. (31)	International; scoping review of 12 studies	Breakfast skipping; anxiety and depression	Consistent association between breakfast skipping and higher anxiety and depression risk	No
Di Nucci et al. (4)	International; scoping review	Sugar-sweetened beverage intake; sleep; mental health	Higher intake co-occurred with sleep disturbance and mental health problems, suggesting a mutually reinforcing triad	No
Lupu et al. (26)	Romania; cross-sectional survey of adolescents	Dietary autonomy, vegetable and sugar-sweetened beverage intake, health behaviors	Older students had greater dietary autonomy but lower vegetable intake; sociodemographic gradients across behaviors	No (autonomy and intake only)
Cisneros-Vásquez et al. (36)	Spain; cross-sectional study of adolescents	Household food insecurity; depression, anxiety, stress (DASS)	Two- to three-fold higher risk of depression, anxiety, and stress symptoms in food-insecure adolescents	No
Rodríguez-Rojo et al. (27)	Spain; cross-sectional, structural equation modeling	Perceived healthy eating; stress management; peer support; positive mental health	Healthy eating related to positive mental health via stress management, moderated by peer support	No (perceived eating only)
Azaiez et al. (39)	Tunisia; cross-sectional, Arabic EDE-Q validation	Disordered eating (EDE-Q); self-esteem; physical activity; BMI	Self-esteem, physical activity, and BMI were the strongest predictors of eating-disorder symptoms	No
Guttersrud et al. (18)	Norway; psychometric study in secondary-school students	Stage-specific critical nutrition literacy scales; Rasch modeling	Critical nutrition literacy can be measured reliably, but behaves differently from functional knowledge and should not be summed with it	Yes (nutrition literacy)
Vaitkeviciute et al. (19)	International; systematic review of adolescents	Food literacy; dietary intake	Association weak and inconsistently demonstrated; few studies used validated literacy measures	Yes (food literacy)
Coughlan et al. (25)	International; scoping review of school-based youth surveys	Mental health literacy measurement instruments	Wide heterogeneity in operationalization, including items adjacent to the nutrition domain; no equivalent mapping exists for nutrition health literacy	No (mental health literacy)
Cohen et al. (45)	International; systematic review and meta-analysis, 75 brief school interventions	Brief school-based mental health or well-being programs	Small but statistically significant effects, sustained at one-year follow-up	No
Wong et al. (11)	International; systematic review, 17 school programs	School-based eating-disorder prevention	10 of 17 programs (59%) improved mental health; dissonance-based and body-acceptance approaches performed best	No
Tajik et al. (32)	Iran; randomized controlled trial, 347 secondary students, 6 months	IMB-model education on diet, activity, stress; knowledge, attitude, behavior; depression	Physical activity increased, unhealthy eating decreased, depression scores fell by 1.9 points at 6 months	Partly (knowledge and attitude, no validated literacy instrument)
Egan et al. (43)	Australia; cluster randomized controlled trial, 71 schools	Health4Life multiple-behavior intervention; socioeconomic status and remoteness as moderators	Weaker dietary improvement in remote than urban areas, indicating the need for tailored designs	No
Brinsley et al. (47)	International; systematic review and meta-analysis, 7 trials	Peer-led lifestyle interventions; psychological distress, self-efficacy, well-being	No significant effects on any mental health outcome	No
Wanjohi et al. (51)	Kenya; longitudinal study of adolescent girls in urban slums (2017–2019)	Anthropometry; double burden of malnutrition	Coexisting wasting and overweight; illustrates the scarcity of longitudinal evidence from low- and middle-income settings	No

Studies are grouped by the pathway or theme they illustrate and are listed in the order of first citation within each theme. The final column records whether nutrition health literacy, or a closely related literacy construct, was itself measured. ARFS, Australian Recommended Food Score; BMI, body mass index; CIS-R, Clinical Interview Schedule–Revised; DASS, Depression Anxiety Stress Scales; EDE-Q, Eating Disorder Examination Questionnaire; GSHS, Global School-Based Student Health Survey; IMB, Information–Motivation–Behavioral Skills; MFQ, Mood and Feelings Questionnaire; PedsQL, Pediatric Quality of Life Inventory.

## 2 Nutrition health literacy: concept, measurement, and correlates

### 2.1 Definition and boundaries with related constructs

Nutrition health literacy is a multidimensional construct spanning functional, interactive, and critical levels (14): obtaining and understanding basic information such as food labels and dietary guidelines; applying it in social settings for communication and decision-making; and analyzing and reflecting on information sources, sociocultural influences, and the food environment (6, 7). Following integrated models of health literacy (5), we define nutrition health literacy as the knowledge, motivation, and competencies to access, understand, appraise, and apply nutrition information for health decisions.

Two boundary distinctions are needed, for different reasons. The distinction from food literacy is definitional: food literacy centers on practical skills such as planning, selecting, and preparing food, and systematic comparison of published definitions shows the terms are used interchangeably far more often than their content warrants (15, 16). The distinction from mental health literacy is of a different kind. As constructs, the two are plainly separate; mental health literacy concerns recognizing, managing, and seeking help for mental health problems (17), and we do not present them as rival definitions. We raise the distinction because they become entangled in practice in exactly the setting this review concerns: school curricula and surveys often address both under a single “health literacy” heading, and items on the diet–emotion link or on recognizing early disordered eating are assigned to one construct or the other depending on the study. Findings generated under one label are therefore liable to be read as evidence for the other, inflating an already thin evidence base.

In adolescents, these competencies are constrained by cognitive maturity, educational background, and social experience. Young people obtain nutrition information mainly from school health education, family elders, social media, and peers, and the accuracy of these sources varies widely, which challenges their ability to evaluate information (6, 7).

### 2.2 Measuring nutrition health literacy in adolescents

Current tools for assessing nutrition health literacy in adolescents lack a unified standard. Some studies use closed-ended scales to measure nutrition knowledge; others combine scenario simulations or open-ended questions to assess critical thinking and decision-making (7, 25). Instrument development has been most systematic at the critical level: stage-specific critical nutrition literacy scales validated by Rasch modeling in secondary-school students measure reliably, but function differently from functional knowledge items and should not simply be summed with them (18). Adolescent instruments nonetheless remain weighted toward functional knowledge.

The measurement boundary is correspondingly unclear. A scoping review of school-based youth surveys documented wide heterogeneity in how mental health literacy is operationalized, including items on early signs of eating disorders and on the diet–emotion link that lie close to the nutrition domain (25). We cite it as evidence of heterogeneity at the interface between the two constructs, not as evidence about nutrition health literacy instruments, which it does not assess. No equivalent mapping exists for adolescent nutrition health literacy measurement—an absence that is itself part of the problem identified here, and the reason work on the adjacent construct must be read with care rather than borrowed.

### 2.3 Correlates of nutrition health literacy

Factors associated with literacy levels, as distinct from the way literacy is measured, form a third strand. Sex, age, socioeconomic status, and urban–rural differences are all associated with literacy levels. A cross-sectional study of Romanian adolescents found that students of different ages differed in dietary autonomy, vegetable intake, and consumption of sugar-sweetened beverages; older students had more autonomy but, paradoxically, lower vegetable intake (26). Since that study measured autonomy and intake rather than literacy, it describes the developmental context in which literacy operates. Improving literacy therefore requires more than knowledge transfer: it must align with adolescents’ developmental characteristics and social environment.

High literacy does not automatically translate into healthy eating, because behavioral decisions are also shaped by emotional state, peer pressure, food availability, and cultural norms (27, 28). A systematic review of food literacy and dietary intake in adolescents found this link weak and inconsistently demonstrated, with few studies using validated literacy measures (19)—the most direct evidence available that literacy is not a sufficient condition for behavior change. Assessment should therefore move beyond simple knowledge testing to incorporate behavioral intention, self-efficacy, and social support.

## 3 Diet, physical activity, and sleep: behavioral pathways to mental health

Dietary behavior is the most extensively studied pathway. Consistent with the framing above, the evidence here concerns diet and mental health directly; nutrition health literacy is a hypothesized upstream influence on dietary patterns, not a measured variable. Substantial cross-sectional evidence shows that unhealthy dietary patterns, high in sugar and fat and low in fruit and vegetables, correlate with depressive and anxiety symptoms (1, 29), and that healthy patterns are linked to lower psychological distress (30). Breakfast regularity is particularly important: a scoping review of 12 studies found a consistent association between skipping breakfast and anxiety and depression (31), and Philippine national data showed that 68.1% of adolescents had skipped at least one meal in the past month, a behavior associated with low fruit and vegetable intake, suicidal ideation, and psychological distress (3).

Cross-sectional designs dominate this literature, and prospective evidence gives a more cautious picture. Earlier systematic reviews found the diet–mental health association consistent in direction but resting overwhelmingly on cross-sectional data, with few studies able to order the variables in time (8, 20). Where cohorts have been followed, results diverge. In an Australian cohort of 3,040 adolescents followed over 2 years, improving diet quality predicted improving psychological functioning, and deterioration predicted the reverse, independently of baseline symptoms (21). In the UK ROOTS cohort, by contrast, diet quality showed neither cross-sectional nor prospective associations with depressive symptoms after adjustment for socioeconomic position, body mass index, and other health behaviors (22). Reverse causation is plausible throughout, since depressive symptoms alter appetite and food choice. The defensible summary is that diet quality and adolescent mental health co-vary robustly, that a diet-to-mood pathway is supported but not securely established, and that no cohort has yet placed nutrition health literacy at the start of that sequence.

Physical activity is a second pathway. Regular exercise helps maintain a healthy weight and benefits mental health by releasing endorphins, improving neurotransmitter balance, and providing opportunities for social interaction (32, 33). Body-image perception complicates this link: among French adolescents with overweight or obesity, boys who underestimated their weight status showed higher energy expenditure from vigorous activity (34). The relationship is also not unidirectional, since depressed mood can itself reduce activity. Prospective accelerometer data from a large UK birth cohort are consistent with effects in both directions: sedentary behavior between ages 12 and 16 predicted higher depressive symptoms at age 18, whereas light activity predicted lower symptoms (23). A randomized controlled trial based on the Information–Motivation–Behavioral Skills model significantly raised physical activity, with depression scores falling by 1.9 points at six-month follow-up (32).

Sleep is a third pathway. Physiological and psychological changes make adolescents especially sensitive to insufficient sleep, and sleep quality is bidirectionally linked to diet. A scoping review found that higher sugar-sweetened beverage intake coexisted with sleep disturbance and mental health problems, forming a potentially mutually reinforcing risk triangle (4). Insufficient sleep affects appetite-regulating hormones such as leptin and ghrelin, and may weaken emotional regulation and increase impulsive and emotional eating (4, 35). Longitudinal data again qualify the direction of effect: in a community adolescent sample, poor sleep quality predicted later anxiety and depression, but baseline symptoms also predicted later sleep problems, consistent with a reciprocal rather than one-way relationship (24). Sleep should therefore be treated as a key mediator or moderator in any analysis of how literacy may relate to mental health.

These behaviors do not act in isolation. A structural equation modeling study found that perceived healthy eating was linked to positive mental health through the mediating role of stress management, with peer support as a moderator (27). Optimizing behavioral pathways therefore requires coordinated change across several lifestyle domains.

## 4 Psychosocial determinants: family, peers, school, and sociocultural context

Parenting style, the home food environment, and parents’ own literacy all have lasting effects on diet and mental health. An Australian study found that adolescent-perceived parental monitoring correlated positively with fruit and vegetable intake, but also with sugar-sweetened beverage consumption (9). Rather than a contradiction, this may reflect that closely monitored adolescents live in homes where engaged parents provide more of both; reverse causation, with parents tightening monitoring in response to a poorer diet, is also plausible. The finding cautions against treating any single parenting behavior as uniformly protective. Among Lebanese adolescents, private-school students showed different dietary patterns from public-school peers, reflecting family socioeconomic status (33). Food insecurity poses a particular threat: Spanish adolescents experiencing it had a two- to three-fold higher risk of depression, anxiety, and stress symptoms (36).

Peers exert growing influence during adolescent socialization, sometimes exceeding that of the family. The transmission of body-image norms, endorsement or rejection of particular diets, and the quality of peer support are all linked to eating-disorder risk and mental health (27, 37). A structural equation modeling study of Jordanian adolescents found that bullying mediated the link between weight status and quality of life, while peer support was protective (38). Individual psychological resources matter alongside these social ones: among Tunisian adolescents, self-esteem, physical activity, and body mass index were the strongest predictors of eating-disorder symptoms (39). That study did not measure nutrition health literacy, and we cite it for what it does establish, that the proximal correlates of disordered eating are psychological and social rather than informational, which is itself a reason not to expect literacy alone to be protective.

Schools are key environments for shaping health behavior, and the content and quality of school health education directly affect literacy. However, a survey in the Philippines found that, although 89.1% of students reported receiving education on healthy eating, meal skipping remained common, suggesting that knowledge transfer alone may be insufficient to change behavior (3). The social–emotional climate, sports facilities, and food-supply policies of a school together form an ecological system affecting diet and mental health (40, 41). In China, the government has improved school sports facilities and food supply to support obesity prevention (40), yet marked inequality in health resources between schools may produce uneven intervention effects.

Sociocultural factors also have a deep influence, including media portrayals of body image, esthetic ideals, and culturally embedded dietary traditions. A study of Chilean adolescents revealed complex associations among body dissatisfaction, eating-disorder risk, and weight status, in which attention to social-media body image was an important variable (42). Among transgender and gender-diverse youth, identity-related stress, medical bias, and the pursuit of gender-affirming appearance shape distinctive dietary attitudes, calling for culturally sensitive and gender-inclusive interventions (10). Socioeconomic inequality is repeatedly confirmed as a key structural factor (43, 44).

## 5 School- and community-based prevention strategies

School-based interventions are widely regarded as among the most accessible and sustainable strategies for improving adolescent diet and mental health, and take many forms: nutrition education, physical activity promotion, social and emotional learning, cognitive-behavioral training, and peer education. A meta-analysis of 75 brief school interventions found small but statistically significant effects on mental health outcomes, persisting at one-year follow-up (45). A systematic review of eating-disorder prevention found that 10 of 17 school programs (59%) improved mental health, with dissonance-based and body-acceptance approaches performing best (11).

Comprehensive lifestyle-education programs show multidimensional benefits. In the Tehran trial noted above, 6 months of education on diet, physical activity, and stress management improved knowledge, attitude, behavior, and depressive symptoms (32), and a quasi-experimental study in Makkah found that a school obesity-control program raised obesity-related knowledge, with lower-knowledge students more prone to moderate-to-severe binge eating (46). These trials are the closest existing analog to a literacy intervention, since they modify knowledge and appraisal and then measure mental health. None, however, used a validated literacy instrument or tested literacy as the mechanism of change, so they support the plausibility of the pathway without evidencing it.

Peer education offers distinct advantages, including use of existing social networks, reduced stigma, and feasible delivery. However, a meta-analysis of seven peer-led lifestyle interventions found no significant effects on psychological distress, self-efficacy, or well-being (47), suggesting that such programs need better educator training, more targeted content, and stronger evaluation. Electronic health interventions show potential in reach and cost-effectiveness, yet a meta-analysis reported that school-based e-mental-health interventions did not significantly improve anxiety or depression, and that study quality was generally low (48).

Prevention design must also account for subgroup needs, since socioeconomic status and geographic location moderate intervention effects: in the Health4Life cluster trial across 71 Australian schools, dietary improvement was weaker among adolescents in remote than urban areas (43). More targeted programs are needed for high-risk populations such as refugee youth, gender-diverse youth, and girls with early menarche (10, 37, 49). Family-based treatment shows promise for loss-of-control eating, with an abstinence rate of 49% immediately post-treatment rising to 73% at 12 months (50).

## 6 Discussion

Although the proposed link between nutrition health literacy and adolescent mental health has attracted considerable interest, the direct evidence remains limited and important controversies persist. The most contested is the effectiveness and possible harm of universal school-based intervention. Some argue that the mixed effects of recent large trials should be read not as failure of universal prevention but as an opportunity to refine design (12). Others warn that non-targeted social and emotional learning may pathologize normal developmental experiences, reinforce self-monitoring, or create cultural mismatch, thereby undermining resilience and generating adolescent resistance and unintended distress (13). The core of the debate is how to balance structured health guidance with respect for adolescent autonomy.

Research gaps are equally notable. First, coverage is highly uneven: most high-quality studies come from high-income countries (41), and work such as the Kenyan longitudinal study of adolescent girls in urban slums, documenting coexisting wasting and overweight, remains rare (51). Second, assessment tools lack a unified standard, and existing scales emphasize functional knowledge while undermeasuring critical literacy and decision-making (18, 25). Third, the cohorts reviewed above show that prospective designs here are feasible (21–24), but they address diet, activity, and sleep rather than literacy: to our knowledge, no longitudinal study has measured nutrition health literacy at baseline and mental health at follow-up, and no trial has tested literacy gain as the mechanism of a mental health effect (1, 31).

A further and central limitation concerns the organizing construct itself. Most available studies measure dietary behavior, food insecurity, self-esteem, or body image in relation to mental health rather than nutrition health literacy directly (Table 1). The proposition that literacy drives these relationships, depicted in Figure 1, therefore remains a conceptual model awaiting direct test with validated instruments and designs that order literacy, behavior, and mental health in time. What limits the field is the absence of such tests rather than negative findings from them. As a focused, recency-weighted mini review without formal quality appraisal, this article is best read as a map of current directions rather than an exhaustive synthesis.

Four priorities follow. First, standardized, multidimensional assessment tools are needed that capture adolescents’ cognitive development and cultural differences. Second, longitudinal and causal-inference designs should be strengthened to clarify bidirectional relationships and identify mediators and moderators; an analysis of the UK Millennium Cohort Study, in which early environmental risk influenced adolescent obesity through childhood mental health problems and later lifestyle factors, illustrates the multi-pathway, multi-timepoint framework this requires (44). Third, intervention research should prioritize cultural adaptation and equity, with programs tested across ethnic, sex, socioeconomic, and geographic groups (10, 43). Fourth, digital and interactive learning methods show promise, but their effectiveness, safety, and accessibility require further study (7, 48).

In conclusion, nutrition health literacy is a plausible and potentially modifiable factor in adolescent mental health, although the current evidence base speaks more directly to diet than to literacy. Its proposed effects would operate through the behavioral pathways of diet, physical activity, and sleep, shaped by family, peer, school, and sociocultural determinants. Evidence consistently links unhealthy dietary patterns to higher risks of depression, anxiety, and eating disorders, with prospective cohorts supporting, but not confirming, a diet-to-mood direction (1, 3, 21, 31). School-based prevention shows modest benefits that vary across subgroups (11, 45), and debate over the value and possible harm of universal intervention calls for critical reflection alongside practical action (12, 13). Nutrition health literacy education should be embedded in school health curricula within a coordinated school–family–society system (40, 41) and designed on the principle of proportionate universalism (12, 43). Strengthening it offers a promising direction for adolescent mental health, one that now warrants direct empirical evaluation.
